# Being ‘in sync’—is interactional synchrony the key to understanding the social brain?

**DOI:** 10.1093/scan/nsaa148

**Published:** 2020-10-26

**Authors:** Annett Schirmer, Merle Fairhurst, Stefanie Hoehl

**Affiliations:** Department of Psychology, The Chinese University of Hong Kong, Shatin, Hong Kong SAR; Brain and Mind Institute, The Chinese University of Hong Kong, Shatin, Hong Kong SAR; Center for Cognition and Brain Studies, The Chinese University of Hong Kong, Shatin, Hong Kong SAR; Institute for Psychology, Bundeswehr University Munich, Munich, Germany; Munich Center for Neuroscience, Ludwig Maximilian University, Munich, Germany; Faculty of Psychology, University of Vienna, Vienna, Austria

**Keywords:** entrainment, rhythm, timing, communication

## Abstract

The past couple of decades produced a surge of interest in interaction synchrony. Moving from the study of behavioral coordination to investigating the coordination of psychophysiological and brain activity, relevant research has tackled a broad range of interactional settings with a multitude of measurement and analysis tools. This method diversity produced a host of interesting results converging on the fact that individuals engaged in social exchange tend to temporally align external as well as internal processes. Moreover, there appears to be a reciprocal relationship between the individuals’ affective bond and the extent of synchronization, which together benefit interaction outcomes. Notably, however, the current breadth of study approaches creates challenges for the field, including how to compare findings and how to develop a theoretical framework that unites and directs ongoing research efforts. More concerted efforts are called for to achieve the conceptual and methodological clarity needed to answer core questions and enabling a balanced pursuit of both synchronous and asynchronous processes.

## Why this special issue?

There are many attributes that differentiate humans from earth’s other life-forms. However, perhaps the most important attribute, allowing humans to thrive in unprecedented ways, is their sociality—that is their urge to aggregate and to organize activities in groups (Dunbar and Shultz, 2007). Recognizing this importance, experimental research moved from the pursuit of mental processes elicited by simple, non-living objects to the study of social perception and cognition (e.g. Bentin *et al.*, 1996; Belin *et al.*, 2002). Moreover, researchers began to examine the human mind in real social interactions (Schilbach *et al.*, 2013) and develop theories that see mental processes as shaped by our species’ social lifestyle and as implemented by neural circuits forming a ‘social brain’ (Adolphs, 2009).

The results of these efforts delineated an interesting phenomenon, namely that humans tend to temporally coordinate when engaging with each other. Also referred to as interactional synchrony, this phenomenon was first empirically documented for non-verbal behavior in the last century. Back then, pioneers in the study of interpersonal processes observed that two individuals in dialogue may mirror each other’s facial expressions or gestures and may move in a manner reminiscent of dancing (Condon and Ogston, 1966; Condon and Sander, 1974). More recently, similar effects were observed in other species (albeit the degree of coordination varies; for a review, see Schirmer *et al.*, 2016) and for non-behavioral measures of human physiology including peripheral (Konvalinka *et al.*, 2011) and central nervous system activity (Reindl *et al.*, 2018). Moreover, rather than being restricted to social interactions, they also emerge in a non-social context, when individuals are exposed to a simple dynamically changing stimulus (Escoffier *et al.*, 2010). Thus, studying the human tendency to synchronize external as well as internal bodily processes seems relevant for us to understand the fundamental mechanisms by which humans engage with both their inanimate and animate environment.

On this backdrop, the present issue of the journal *Social Cognitive and Affective Neuroscience* brings together an exciting set of articles that tackle synchrony emerging in social but also non-social settings. These articles comprise a collection of empirical and theoretical contributions shedding light on a wide range of study methodologies, findings and opinions. Together, they offer a snapshot of the current standing of the field and identify both its opportunities and challenges.

### A context for synchrony

The present collection of articles covers a wide range of synchronizing situations. Some of these situations are very typical of everyday life, including interactions we have with household pets (Axelsson and Fawcett, this issue), friends (Bolis *et al.*, this issue) or family members (Nguyen *et al.*, this issue), whereas others are more exceptional like a joint musical performance of trained musicians (Zamm *et al.*, this issue). What these situations reveal is that synchrony depends on whether individuals have a special connection or bond. Much evidence suggests that such a bond can facilitate the temporal alignment between interaction partners. For example, synchrony is more readily observed when children interact with their parents as compared to unfamiliar adults (Reindl *et al.*, 2018), when women solve a problem together with other women as compared to men (Thorson and West, 2018) or when two unfamiliar individuals find each other attractive and are interested in a date (Chang *et al.*, this issue). Another factor influencing interpersonal synchrony may be personality similarities and differences between interaction partners (Bolis *et al.*, this issue).

Notably, however, a special connection or bond is not necessary for synchrony to emerge. It seems instead that this merely facilitates attention or positive engagement with a social or non-social stimulus. Indeed, the wish to cooperate with (Reinero *et al.*, this issue; Sciaraffa *et al.*, this issue), to deceive or to detect deception in an unknown partner (Pinti *et al.*, this issue) also prompts interactional synchronizing. Moreover, the mere exposure to regularly repeating sounds amplifies relevant sound frequencies in the brain and benefits the cyclic allocation of attention irrespective of modality (Schirmer *et al.*, this issue). Thus, the brain constantly picks up on temporal regularities in an individual’s environment. Yet, individuals synchronize selectively depending on motivational factors and top-down processes of dynamic attention allocation (Hoehl *et al.*, this issue).

While aspects of the context or situation modulate the extent to which individuals regulate the timing of external and internal bodily processes, effects are not unidirectional. Indeed, the presence or absence of synchrony may shape ongoing exchanges, thus promoting or impairing, respectively, the likelihood of positive individual and social outcomes (Reinero *et al.*, this issue). Causal evidence for this was obtained by inducing or disrupting brain synchrony using transcranial alternating current stimulation (tACS). Compared with the latter, the former condition facilitated movement alignment between teacher and students, which partially mediated enhanced learning (Pan *et al.*, this issue).

### Methods for studying synchrony

Today’s synchrony research is characterized by a broad method spectrum, with some attempts to systematize different approaches (Levy *et al.*, 2017; Misaki *et al.*, this issue). Indeed, there is much variation in study paradigms, measurement variables and analytical strategies that is reflected in the current issue.

Paradigms differ in whether they elicit intentional or unintentional synchronization. Music making or tapping are examples for the former approach (Heggli *et al.*, this issue; Zamm *et al.*, this issue) and passively observing others (Kragness and Cirelli, this issue) or engaging in conversation (Nguyen *et al.*, this issue; Thorson *et al.*, this issue) are examples for the latter approach. As mentioned above, some authors even attempted to induce synchrony through brain stimulation (Pan *et al.*, this issue).

Existing data types can be classified by whether they concern observable behavior or internal nervous system activity. Moreover, each of these measurement types can be further differentiated. Behavioral research may focus on a specific expressive feature such as body sway (Chang *et al.*, this issue) or consider any kind of motion. The study of internal parameters might focus on the peripheral nervous system with measures such as skin conductance (Kragness and Cirelli, this issue) or heart rate (Thorson *et al.*, this issue) or on brain changes with neuroimaging measures. Interestingly, the latter is pursued most frequently not with functional magnetic resonance imaging, which many consider the gold standard of neuroimaging, but with other less popular techniques including the electroencephalography (Heggli *et al.*, this issue; Schirmer *et al.*, this issue; Zamm *et al.*, this issue), magnetoencephalography (Levy *et al.*, 2017) and functional near-infrared spectroscopy (Dieffenbach *et al.*, this issue; Kruppa *et al.*, this issue; Nguyen *et al.*, this issue; Pinti *et al.*, this issue). This choice nicely reflects the need to capture the fast temporal dynamics characterizing interactional synchrony and/or the need for fairly unconstrained face-to-face interactions (but see Misaki *et al.*, this issue).

Irrespective of measurement types or techniques, synchrony research must address the problem of how to relate two or more recorded time series. To this end, many mathematical approaches have been developed and applied. Some of these approaches, featuring in the current issue, include simple cross-correlations of original or wavelet-transformed time series (Kruppa *et al.*, this issue; Pan *et al.*, this issue) as well as indices of phase locking (Heggli *et al.*, this issue) or shared changes in the power of certain frequencies characterizing the time series (Schirmer *et al.*, this issue; Zamm *et al.*, this issue). These as well as other analytical strategies including Granger causality (Sciaraffa *et al.*, this issue) differently address the problem of how to define synchrony and whether and how delays between corresponding changes in the time series of interest (e.g. leads/lags) should be considered and mathematically modeled. This is especially important as differences in the alignment of two time series may be of functional significance (Jiang *et al.*, this issue).

### Opportunities and challenges

Undoubtedly, it has been beneficial for synchrony research to expand its original focus on non-verbal expressions to the present multitude of paradigms, measures and analysis strategies. However, the current methodological breadth also creates challenges and calls for efforts to consolidate and converge on a set of central theoretical questions and the best way to address them. The creative burst unfolding in the last decades must be followed by a more strategic pruning and organization of research programs.

Towards this end, the present collection of papers makes an initial effort by providing insightful reviews of study approaches and findings (Hoehl *et al.*, this issue; Jiang *et al.*, this issue; Levy *et al.*, this issue; Misaki *et al.*, this issue), empirically linking different synchrony measures (Pan *et al.*, this issue; Sciaraffa *et al.*, this issue) and by introducing a new python-based software package that can help with standardizing data analysis (Ayrolles *et al.*, this issue). However, more work is needed. Indeed one would hope for better concerted efforts to define synchrony or to specify different forms of synchrony.

One interesting issue here is whether and to what extent we should consider the alignment of temporal patterns from different sources. This seems a critical step in analyzing the coordination of neural activity between individuals, for instance, in verbal conversation, when neural activity in auditory cortices of the listener aligns with activity in articulatory motor areas of the speaker (Jiang *et al.*, this issue). But how meaningful is it for other source combinations (e.g. brain/heart, heart/behavior)?

Another interesting issue arises from the fact that synchrony is often explicitly and implicitly linked to rhythms. However, rhythms or oscillatory activity is not necessary for synchronizing to occur (Schirmer *et al.*, this issue). Apart from music making, social interactions are not strictly sinusoidal or metrical but, as has been reviewed here, can still be temporally predictable and synchronizing. Thus, metrical or oscillatory processes need to be differentiated from non-oscillatory ones.

An important future direction will be to reign in the current enthusiasm for discovering synchrony and to start thinking more carefully about its shades and functions. Although synchrony can be beneficial, like all things in life it may be too much of a good thing. Moreover, current approaches look at synchrony in a fairly superficial manner by, for example, comparing the mean values obtained in one condition to those from another condition. However, like its underlying time series measures, synchrony is a dynamical phenomenon that likely waxes and wanes, breaks and resets (Likens and Wiltshire (n.d)). Hence, moments of low or no synchrony may be as or perhaps more important than those with high synchrony.

With this in mind, we see this special issue as an important milestone in our quest to understand the biological basis of interactional synchrony and its role in human social exchanges. But more importantly, we think that the manuscripts collected here delineate important future directions and hope they provide the field with the necessary momentum for new and fundamental discoveries.
